# Prognostic Factors and Scoring System for Death from Visceral Leishmaniasis: An Historical Cohort Study in Brazil

**DOI:** 10.1371/journal.pntd.0003374

**Published:** 2014-12-11

**Authors:** Wendel Coura-Vital, Valdelaine Etelvina Miranda de Araújo, Ilka Afonso Reis, Frederico Figueiredo Amancio, Alexandre Barbosa Reis, Mariângela Carneiro

**Affiliations:** 1 Pós-graduação em Ciências da Saúde, Infectologia e Medicina Tropical, Faculdade de Medicina, Universidade Federal de Minas Gerais, Belo Horizonte, Minas Gerais, Brasil; 2 Núcleo de Pesquisas em Ciências Biológicas, Instituto de Ciências Exatas e Biológicas, Universidade Federal de Ouro Preto, Ouro Preto, Minas Gerais, Brasil; 3 Secretaria Municipal de Saúde, Prefeitura de Belo Horizonte, Belo Horizonte, Minas Gerais, Brasil; 4 Departamento de Parasitologia, Instituto de Ciências Biológicas, Universidade Federal de Minas Gerais, Belo Horizonte, Minas Gerais, Brasil; 5 Departamento de Estatística, Instituto de Ciências Exatas, Universidade Federal de Minas Gerais, Belo Horizonte, Minas Gerais, Brasil; 6 Fundação Hospitalar do Estado de Minas Gerais, Belo Horizonte, Minas Gerais, Brasil; Universidade Federal de Minas Gerais, Brazil

## Abstract

**Background:**

In Brazil, case-fatality rates attributable to visceral leishmaniasis (VL) are high and knowledge of the risk factors associated with death may help reduce mortality. The aim of this study was to construct and validate a scoring system for prognosis of death from VL by using all cases reported in Brazil from 2007 to 2011.

**Methodology:**

In this historical cohort study, 18,501 VL cases were analyzed; of these, 17,345 cases were cured and 1,156 cases caused death. The database was divided into two series: primary (two-thirds of cases), to develop the model score, and secondary (one-third of cases), to validate the scoring system. Multivariate logistic regression models were performed to identify factors associated with death from VL, and these were included in the scoring system.

**Principal Findings:**

The factors associated with death from VL were: bleeding (score 3); splenomegaly (score 1); edema (score 1); weakness (score 1); jaundice (score 1); *Leishmania*–HIV co-infection (score 1); bacterial infection (score 1); and age (≤0.5 years [score 5]; >0.5 and ≤1 [score 2]; >19 and ≤50 [score 2]; >50 and <65 [score 3]; ≥65 [score 5]). It was observed that patients with a score of 4 had a probability of death of approximately 4.5% and had a worse prognosis. The sensitivity, specificity, and accuracy of this score were 89.4, 51.2, and 53.5, respectively.

**Conclusions/Significance:**

The scoring system based on risk factors for death showed good performance in identifying patients with signs of severity at the time of clinical suspicion of VL and can contribute to improving the surveillance system for reducing case fatalities. The classification of patients according to their prognosis for death may assist decision-making regarding the transfer of the patients to hospitals more capable of handling their condition, admission to the intensive care unit, and adequate support and specific treatment.

## Author Summary

Visceral leishmaniasis (VL) is a fatal disease if not diagnosed and treated appropriately. In the present study, we investigated the risk factors associated with death caused by VL identifiable at the time of clinical suspicion. This study was conducted using all VL cases registered in Brazil during 2007 to 2011. The prognostic factors associated with death caused by VL were bleeding, age (1 year or younger and older than 19 years), *Leishmania*–human immunodeficiency virus (HIV) co-infection, bacterial infection, splenomegaly, edema, weakness, and jaundice. A prognostic scoring system for death caused by VL ranging from 0 to 14 was developed and validated using these risk factors. Patients with scores of 4 or more presented with a worsening prognosis. The scoring system showed good performance in identifying the severe cases. Classification of patients according to their prognosis for death can contribute to improving the clinical management of severe cases and reducing case-fatality rates associated with VL.

## Introduction

Visceral leishmaniasis (VL) is a severe chronic systemic disease caused by *Leishmania infantum* parasites in South America, the Mediterranean region, and southwest and central Asia [1]. The disease is transmitted to human and animal hosts by the bite of phlebotomine sand flies, and dogs are the main urban reservoirs [2], [3]. VL is clinically characterized by prolonged fever, weakness, anorexia, weight loss, hepatomegaly, splenomegaly, hypergammaglobulinemia, and pancytopenia. Over time, without treatment, the disease may progress to severe cachexia, multisystem disease, bleeding, secondary infections, and death [4], [5]. Bacterial infections and bleeding have been the two most prominent symptoms associated with death caused by VL [6].

In Brazil, the occurrence of VL was initially limited to rural areas and small urban locations, but in past decades it has expanded into large urban centers and has become an increasing public health problem throughout the country [7], [8]. Autochthonous cases were recorded in 26 of the 27 states of Brazil, indicating the dispersion of the disease throughout the country. From 2001 to 2011, 39,780 confirmed VL cases had been reported in Brazil, with an annual average of 3,616 new cases, incidence rate of 2.0 cases per 100,000 inhabitants, and case-fatality rate of 6.5% during this period [9], [10].

Although more specific guidelines for the management of patients with severe VL have been developed in Brazil, the case-fatality rate remains high [9], [11], [12]. To reduce mortality, the Brazilian Ministry of Health has instituted specific recommendations through the Visceral Leishmaniasis Control and Surveillance Program (VLCSP). These guidelines include early diagnosis and treatment of human cases, vector control, serological screening and subsequent culling of infected dogs, and health education [11]. According to the VLCSP, all suspected and confirmed cases of VL must be reported to the sanitary authorities and registered in the Reportable Disease Information System (SINAN). This system provides a center for the collection and processing of data and helps to disseminate information generated by the epidemiological surveillance systems linked to the municipal, state, and federal governments. Moreover, this information system contributes to the knowledge of the worldwide morbimortality caused by VL because it helps consolidate the data from institutions such as the Pan American Health Organization and World Health Organization, as recently reported by Alvar et al. [13].

Because reduction of the case-fatality rate is one of the goals of the disease control program, it is important to study the factors associated with death caused by VL. Furthermore, considering that most deaths attributable to VL occur in poor countries [8], the development of a prognostic score using clinical parameters and the dispensing of laboratory results are extremely important. This scoring system can help reduce fatality rates.

Some studies have been performed to propose a prognostic scoring system for death in Brazil, but they used different sources of data to analyze specific age groups or regions of the country [14]–[16]. The aim of the present study was to identify risk factors associated with death and propose a prognostic scoring system for death by using an historical cohort study including all human VL cases registered in the Brazilian Reportable Disease Information System during 2007 to 2011. The scoring system for death was proposed to assist in early identification of patients at higher risk for death from VL and to guide more focused strategies to improve the clinical management of cases and reduce the case-fatality rate.

## Methods

### Ethical statement

Data analyzed in the present study were provided by the Department of Health Surveillance of Brazilian Ministry of Health (SINAN/CIEVS/DEVEP/MS). The data were anonymous and did not include information that would allow the identification of individuals or that could affect the confidentiality of data. This study included only public secondary data stripped of any individual identifiers. Therefore, ethics committee approval was not required.

### Study design and population

The historical cohort study was conducted in Brazil and included all new cases of VL registered in the SINAN between January 1, 2007 and December 31, 2011. Brazil has an area of 8,515,767.049 km^2^ and comprises almost half of South America. According to the census by the Brazilian Institute of Geography and Statistics in 2010, the country's population at that time was 190,732,694 inhabitants [10].

The selection criteria for inclusion in the study were as follows: the patient represented a VL case with evolution to cure; the patient represented a VL case with evolution to death; and the primary cause of death of the patient was VL. The exclusion criteria were: no case of VL (ruled out by specific laboratory examinations) or absence of this information (missing); progression to death attributable to other causes; abandonment of treatment; moved to another region during treatment (transference); or absence of this information (missing). As recommended by Snee [17], when collection of new data to validate a model was not possible, simulation of new data was necessary. Splitting the data into two datasets is a reasonable way to accomplish this. The use of one-third of the dataset for validation is necessary because most of the data (two-thirds) must be used to adjust the model to improve the estimation of the parameters [18]. In this sense, the database was randomly subdivided into two series: primary series of patients (two-thirds of eligible cases), which was used to develop the model prognostic score system, and secondary series of patients (one-third of eligible cases), which was used to validate the scoring system. To ensure that the proportion of cures and deaths remained the same in both series, patients were initially divided into two datasets (cures and deaths) ordered by date of notification. In each dataset, a column with the patient's position was generated. For the dataset of cures, 11,563 random numbers between 1 and 17,345, corresponding to two-thirds of the total, were generated. The patients corresponding to the numbers generated were selected for the primary series and the remaining patients were included in the secondary series. The same procedure was used to select patients in the database of deaths, with the generation of 770 random numbers between 1 and 1,156.

### Variables studied

The epidemiological surveillance system of Brazil has recorded the suspected cases of VL in SINAN using a form comprising the following information: date of notification; health unit responsible for care and notification; address; age; sex; level of schooling; occupation of patient; date of the start of symptoms; clinical manifestations (signs and symptoms); co-infections; results of specific laboratory examinations (diagnostic tests); date of beginning of treatment; initial drug used for treatment; drug used after failure of the initial therapy; and evolution of the case. The variables analyzed in the present study were sex and age of patient, date of notification, date of the start of symptoms, clinical manifestations, co-infections (human immunodeficiency virus [HIV]), and evolution of the case (cure or death caused by VL).

### Statistical analysis

Statistical analyses of the data were performed using STATA version 11.0 software (Stata Corp., College Station, TX, USA). To build the scoring system, two-thirds of patients eligible for the study were selected randomly. Univariate logistic regression analysis was used to evaluate the clinical variables according to the occurrence of death from VL. Variables associated with death from VL with a significance level of p<0.25 were included in multivariate logistic regression analyses. Variables with more than two categories, such as age, race, and education, were transformed into dummy variables.

Variables presenting statistical significance but with either collinearity or low frequency were excluded from the multivariate analysis. A step-by-step backward selection procedure was used to select the variables and to produce the final multivariate logistic regression models. Only adjusted variables showing a significant association (p<0.05) with the occurrence of death from VL remained in the final model. The likelihood ratio tests were used to adjust these models. The strength of association was determined by odds ratio (OR) with a 95% confidence interval (CI). The predictive factors of death from VL were used to create a prognosis score. According to the methodology described by Barquet et al. [19], to define the score of each predictive factor, the coefficients of the logistic regression were standardized. Each regression coefficient was divided by the smallest one, and the quotient was rounded to the nearest integer to facilitate clinical use of the system. To estimate the probability of death for the individual *i* (p_i_), the following expression was used:
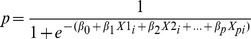
where *β*_0_, *β*_1_,…, *β*_p_ are the original estimates for the coefficients of the logistic regression and *X*1_i_, *X*2_i_,…, *X*p_i_ are the values for the explicative variables of the individual *i*.

The scoring system for prognosis of death was validated using a validation dataset that included one-third of the eligible patients. The actual evolution of each case, defined as death or cure from VL, was compared with the predictive score. The predictive performance of the scoring system was determined by sensitivity, specificity, accuracy, positive predictive values, negative predictive values and the area under the receiver operating characteristic curve (ROC) [20].

To evaluate the possible existence of differences between cases included (18,501) and cases not included (3,576 cases without final classification) in the study, these two groups were compared according to the variables available in SINAN by using the chi-square test.

## Results

**Figure 1:**
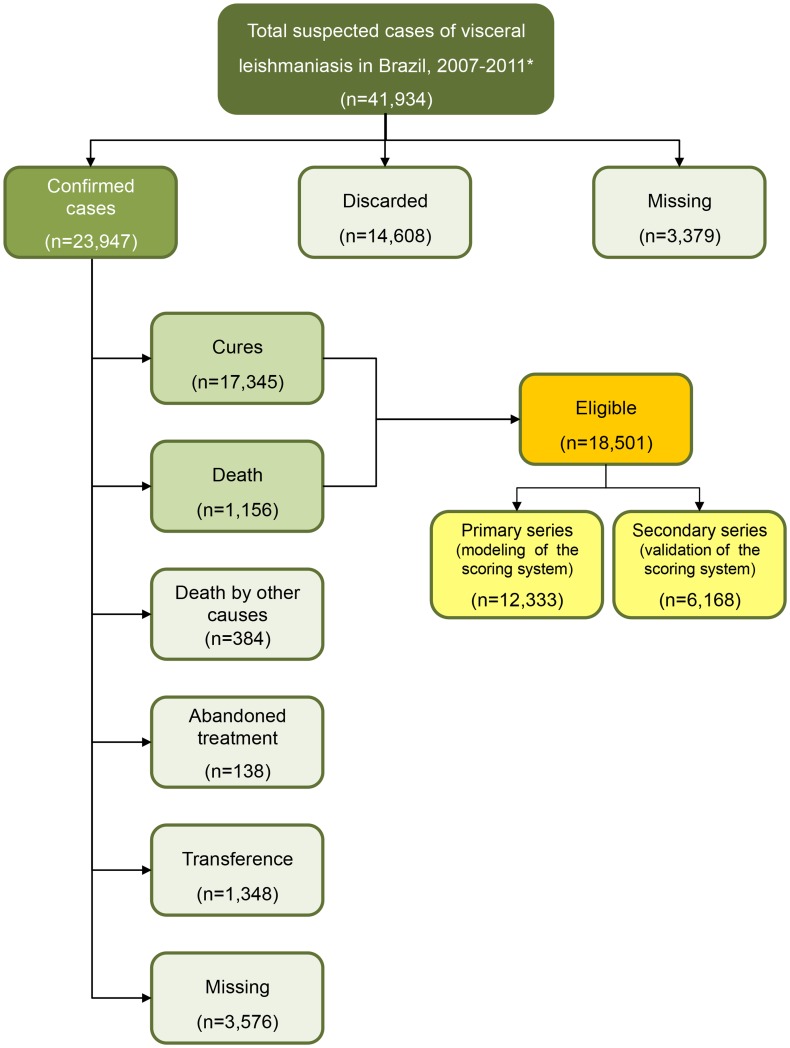
Flow diagram of the population evaluated. *Cases reported in Brazilian Reportable Disease Information System.

A total of 41,934 suspected VL cases were registered in SINAN from 2007 to 2011; of these, 23,947 were confirmed. Among the patients with confirmed cases, 17,345 cases were cured (72.5%) and 1,156 (4.8%) patients died from VL, resulting in 18,501 individuals eligible for analysis. Among the ineligibles cases were 384 (1.6%) patients who died because of other causes, 138 (0.6%) patients who abandoned treatment, 1,348 (5.6%) patients who were moved to another region during the treatment (transference), and 3,576 (14.9%) patients without data regarding the evolution of the case (missing data). Furthermore, among the suspected cases, 14,608 (34.8%) were not included because infection by *L. infantum* was not confirmed and 3,379 (8.0%) had missing information regarding final classification (discarded or confirmed as VL) (Fig. 1).

The chi-square test signaled possible differences (p<0.05) between cases included in and excluded from the study regarding the variables of race, area (urban or rural), and presence of fever and other infections. However, we observed that the categories of the variables have similar proportions (maximum difference of 3%).

### Characteristics of the patients

The characteristics of patients with VL evaluated in the primary series and in the secondary series are shown in Table 1. It is noteworthy that all these characteristics were recorded at the time of clinical suspicion of VL. Significant differences between patients in both series were not observed. Among the patients included in the study, there was a predominance of the following characteristics: male sex; age 6 months to 5 years; time to clinical diagnosis ≤15 days; mulatto race; elementary schooling; and residence in an urban area. The main clinical manifestations were fever, weakness, splenomegaly, weight loss, and pallor. Approximately 7% of patients had *Leishmania*–HIV co-infection (Table 1).

**Table 1:** Demographics and clinical characteristics of patients with visceral leishmaniasis according to primary and secondary series, Brazil 2007–2011.

Variable	Primary Series* n = 12,333	Secondary Series# n = 6,168	*P*
	n (%)	n (%)	
Male	7,613 (61.7)	3,746 (60.7)	0.19
Age (years)			
≤0.5	252 (2.0)	138 (2.2)	
>0.5 to ≤1	682 (5.5)	301 (4.9)	
>1 to ≤5	3,410 (27.7)	1,758 (28.5)	
>5 to ≤19	2,564 (20.8)	1,239 (20.1)	
>19 to ≤50	3,774 (30.6)	1,945 (31.5)	
>50 to <65	1,044 (8.5)	515 (8.4)	
≥65	607 (4.9)	272 (4.4)	0.14
Time to clinical diagnosis (days)**			
≤15	5,101 (41.4)	2,482 (40.2)	
>15 to ≤30	2,553 (20.7)	1,282 (20.8)	
>30 to ≤60	2,219 (18.0)	1,157 (18.8)	
>60	2,460 (19.9)	1,247 (20.2)	0.43
Race			
White	2,311 (20.8)	1,116 (20.1)	
Black	912 (8.2)	505 (9.1)	
Asian	106 (1.0)	56 (1.0)	
Mulatto	7,652 (69.1)	3,817 (68.9)	
Indigenous	97 (0.9)	50 (0.9)	0.34
Education			
College	130 (1.4)	61 (1.3)	
High school	803 (8.3)	407 (8.4)	
Elementary school	2,058 (21.4)	1,019 (21.1)	
Illiterate	1,591 (16.5)	770 (16.0)	
Not applicable (<5 years)	5,048 (52.4)	2,569 (53.2)	0.85
Area			
Urban	9,302 (77.7)	4,667 (77.9)	
Rural	2,664 (22.3)	1,325 (22.1)	0.82
Clinical manifestation			
Fever	11,040 (92.3)	5,547 (92.4)	0.82
Weakness	9,398 (79.8)	4,757 (80.6)	0.22
Edema	2,703 (23.9)	1,353 (23.9)	0.96
Weight loss	8,489 (72.3)	4,270 (72.5)	0.82
Cough and/or diarrhea	5,440 (46.7)	2,763 (47.4)	0.37
Pallor	8,085 (70.5)	4,096 (71.3)	0.30
Splenomegaly	8,754 (75.1)	4,391 (75.4)	0.63
Bacterial infection	2,600 (23.6)	1,287 (23.5)	0.78
Bleeding	1,026 (9.2)	516 (9.2)	0.93
Hepatomegaly	7,663 (66.3)	3,846 (66.6)	0.71
Jaundice	2,500 (22.2)	1,295 (23.0)	0.27
Other manifestations	2,245 (21.0)	1,221 (20.8)	0.77
*Leishmania*–HIV co-infection	610 (7.0)	295 (6.8)	0.58

*Group used to develop the scoring system.

# Group used to validate the scoring system.

**Time between the dates of onset of symptoms and notification (days). HIV, human immunodeficiency virus.

### Modeling the prognostic scoring system (primary series)

The variables obtained by the univariate logistic regression analysis of VL patients were used for modeling the system score (primary series) according to outcome (cure or death) and are shown in Table 2.

**Table 2:** Univariate analysis of the prognostic factors for death in visceral leishmaniasis, Brazil 2007–2011.

Variable	Category	Deaths n = 770	Cures n = 11,563	OR (95% CI)	*p*
		n (%)	n (%)		
Sex	Female	263 (34.2)	4,457 (38.5)		
	Male	507 (65.8)	7,106 (61.5)	1.2 (1.0–1.4)	0.01
Age (years)	>1 to ≤19	167 (21.7)	5,807 (50.2)		
	≤0.5	35 (4.6)	217 (1.9)	5.6 (3.8–8.3)	0.00
	>0.5 to ≤1	55 (7.1)	627 (5.4)	3.1 (2.2–4.2)	0.00
	>19 to ≤50	265 (34.4)	3,509 (30.4)	2.6 (2.2–3.2)	0.00
	>50 to **<**65	121 (15.7)	924 (8.0)	4.6 (3.6–5.8)	0.00
	≥65	127 (16.5)	480 (4.1)	9.2 (7.2–11.8)	0.00
Time to clinical diagnosis (days)*	≤15	254 (33.0)	4,847 (41.9)		
	>15 to ≤30	152 (19.7)	2,401 (20.8)	1.2 (0.9–1.5)	0.07
	>30 to ≤60	138 (17.9)	2,081 (18.0)	1.3 (1.0–1.6)	0.03
	>60	226 (29.4)	2,234 (19.3)	1.9 (1.6–2.3)	0.00
Fever	No	44 (6.0)	872 (7.8)		
	Yes	684 (94.0)	10,356 (92.2)	1.3 (0.9–1.8)	0.09
Weakness	No	73 (10.2)	2,305 (20.8)		
	Yes	642 (89.8)	8,756 (79.2)	2.3 (1.8–3.0)	0.00
Edema	No	377 (55.0)	8,225 (77.5)		
	Yes	308 (45.0)	2,395 (22.5)	2.8 (2.4–3.3)	0.00
Weight loss	No	140 (20.0)	3,109 (28.2)		
	Yes	560 (80.0)	7,929 (71.8)	1.6 (1.3–1.9)	0.00
Cough and/or diarrhea	No	327 (46.4)	5,891 (53.8)		
	Yes	378 (53.6)	5,062 (46.2)	1.3 (1.2–1.6)	0.00
Pallor	No	140 (20.4)	3,237 (30.0)		
	Yes	548 (79.6)	7,537 (70.0)	1.7 (1.4–2.0)	0.00
Splenomegaly	No	112 (15.8)	2,792 (25.5)		
	Yes	598 (84.2)	8,156 (74.5)	1.8 (1.5–2.2)	0.00
Bacterial infection	No	355 (53.5)	8,044 (77.8)		
	Yes	309 (46.5)	2,291 (22.2)	3.0 (2.6–3.6)	0.00
Bleeding	No	448 (66.8)	9,745 (92.4)		
	Yes	223 (33.2)	803 (7.6)	6.0 (5.0–7.2)	0.00
Hepatomegaly	No	155 (21.7)	3,743 (34.5)		
	Yes	561 (78.3)	7,102 (65.5)	1.9 (1.6–2.3)	0.00
Jaundice	No	411 (60.8)	8,331 (78.9)		
	Yes	265 (39.2)	2,235 (21.1)	2.4 (2.0–2.8)	0.00
*Leishmania*–HIV co-infection	No	463 (86.9)	7,586 (93.3)		
	Yes	70 (13.1)	540 (6.7)	2.1 (1.6–2.8)	0.00

*Time between the dates of onset of symptoms and notification (days); CI, confidence interval; HIV, human immunodeficiency virus; OR, odds ratio.

Table 3 shows the following predictors of death from VL identified by the multivariate logistic regression analysis: splenomegaly (OR 1.5; 95% CI 1.2–2.0); edema (OR 1.8; 95% CI 1.4–2.2); weakness (OR 1.7; 95% CI 1.2–2.3); bleeding (OR 3.8; 95% CI 3.0–4.8); jaundice (OR 1.6; 95% CI 1.3–2.0); *Leishmania*–HIV co-infection (OR 1.6; 95% CI 1.2–2.2); bacterial infection (OR 1.9; 95% CI 1.5–2.3); and age (≤0.5 years [OR 8.6; 95% CI 5.3–13.8]; >0.5 to ≤1 year [OR 2.8; 95% CI 1.8–4.3]; >19 to ≤50 years [OR 2.4 95% CI 1.8–3.1]; >50 to <65 years [OR 4.0; 95% CI 2.9–5.7]; and ≥65 years [OR 9.6; 95% CI 6.7–13.6]).

**Table 3:** Predictive scoring system for death attributable to visceral leishmaniasis, Brazil 2007–2011.

Variable	Adjusted OR (95% CI)	Regression Coefficient	Standard Error	Points#
Splenomegaly	1.5 (1.2–2.0)	0.43	0.14	1
Edema	1.8 (1.4–2.2)	0.57	0.11	1
Weakness	1.7 (1.2–2.3)	0.50	0.17	1
Bleeding	3.8 (3.0–4.8)	1.34	0.12	3
Jaundice	1.6 (1.3–2.0)	0.48	0.11	1
Age (years)				
≤0.5	8.6 (5.3–13.8)	2.14	0.24	5
>0.5 to ≤1	2.8 (1.8–4.3)	1.02	0.23	2
>1 to ≤19	1.0	-	-	0
>19 to ≤50	2.4 (1.8–3.1)	0.87	0.14	2
>50 to <65	4.0 (2.9–5.7)	1.34	0.17	3
≥65	9.6 (6.7–13.6)	2.25	0.18	5
*Leishmania*–HIV co-infection	1.6 (1.2–2.2)	0.50	0.16	1
Bacterial infection	1.9 (1.5–2.3)	0.63	0.11	1

Constant of the model = −5.12 and standard error = 0.20.

# Each coefficient was divided by 0.43 and the quotient was rounded to the nearest integer to determine the number of points assigned to that predictor of death from visceral leishmaniasis.

CI, confidence interval; HIV, human immunodeficiency virus; OR, odds ratio.

On the basis of the logistic regression coefficients, one point was assigned for splenomegaly, edema, weakness, jaundice, *Leishmania*–HIV co-infection, and bacterial infection. Three points were assigned for bleeding. In addition, two points were assigned for children older than 6 months and up to 1 year of age and for adults between 19 and 50 years of age; three points were assigned for age range older than 50 years to younger than 65 years. Five points were assigned for children younger than 6 months and also for adults 65 years of age or older (Table 3). The prediction score ranged from 0 to 14; however, no evaluated patient obtained a score more than 13.

### Probability of death

**Figure 2:**
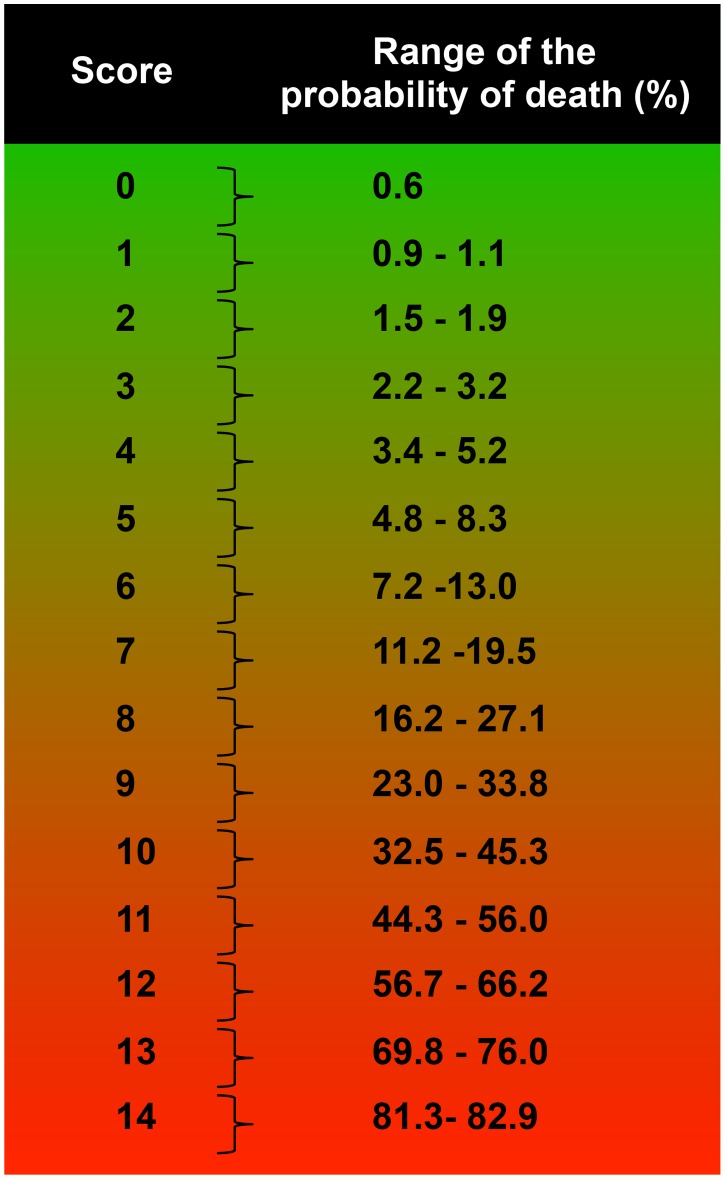
Prognostic scoring system and range of probability of death from visceral leishmaniasis.

The range of probability of death according to the prognostic score of the patients included in the primary series is presented in Fig. 2. Because the same score can be obtained with different prognostic factors (coefficients of the regression), a range of probability of death was defined. These ranges were calculated based on the sum of the smallest and largest regression coefficients for each point comprising the score. For example, for a score of 4, the range of probability of death was calculated as follows: 
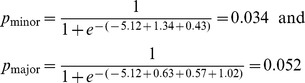
The idea behind the presentation of a range of probability is that a patient can have a given value for the score in different ways. For example, a patient older than 1 year or younger than 20 years can have a score of 4 if that patient presents with splenomegaly and bleeding (p_minor_), or if the patient is older than 0.5 years or younger than 1 year and presents with bacterial infection and edema, (p_major_). The standardization of the coefficients gives the score of 4 for both situations. However, the probability of death is calculated based on the original coefficients, which leads to some difference in values. A score of 3 or less corresponds to a probability of death of less than 3.2%. It is noteworthy that a patient with a score of 4 has approximately 4.5% probability of death from VL, which is relevant from a clinical point of view. The ranges of probability of death are 4.8% to 8.3% and 7.2% to 13.0% when the score is 5 and when the score is 6, respectively. When the patient has a score of 11, the range of probability of death increases to 44.3% to 56.0%; with a score of 14, the range of probability of death is 81.3% to 82.9% (Fig. 2).

### Validation of the prognostic scoring system (secondary series)

The reliability and discriminatory power of the models were good. The lowest scores showed high sensitivity and low specificity, and the opposite was observed for higher scores. The lethality observed in the validation group (secondary series) was similar to probability of death estimated by the score and did not vary by many percentage points, with the exception of scores 11, 12, and 13. Miscalibrations for patients with predicted low risk and high risk during the internal validation were observed. These miscalibrations might be related to the imprecision attributable to the low number of patients in the lowest risk group and in the highest risk group. It was also observed that a score of 4 showed a slightly higher than expected probability of mortality. The sensitivity, specificity, and accuracy of a score of 4 were 89.4, 51.2, and 53.5 for primary series and 86.6, 51.2 and 53.2 for secondary series respectively (Table 4).

**Table 4:** Performance of the prognostic scoring system for death attributable to visceral leishmaniasis, Brazil 2007–2011.

Prediction Score	Evolution*		Observed LethalityRate* (%)	Primary series			Secondary series		
	Cure n (%)	Death n (%)		Sensitivity	Specificity	Accuracy	Sensitivity	Specificity	Accuracy
0	96 (2.6)	1 (0.4)	1.0	100	0	6.0	100	0	5.7
1	376 (10.1)	1 (0.4)	0.3	99.8	2.4	8.2	99.6	2.6	8.1
2	782 (21.1)	11 (4.9)	1.4	98.5	13.1	18.2	99.1	12.7	17.6
3	644 (17.4)	17 (7.6)	2.6	95.4	33.2	36.9	94.2	33.8	37.2
4	669 (18.0)	30 (13.5)	4.3	89.4	51.2	53.5	86.6	51.2	53.2
5	447 (12.0)	37 (16.6)	7.6	78.9	69.5	70.0	73.1	69.2	69.5
6	311 (8.4)	30 (13.5)	8.8	61.7	81.9	80.7	56.5	81.3	79.9
7	182 (4.9)	31 (13.9)	14.5	47.2	89.4	86.8	43.1	89.7	87.0
8	101 (2.7)	19 (8.5)	15.8	32.1	94.7	90.9	29.2	94.6	90.9
9	47 (1.3)	13 (5.8)	21.6	20.1	97.7	93.0	20.6	97.3	93.0
10	40 (1.1)	16 (7.2)	28.6	12.1	99.0	93.8	14.8	98.6	93.8
11	7 (0.2)	10 (4.5)	58.8	5.7	99.7	94.0	7.6	99.7	94.4
12	4 (0.1)	4 (1.8)	50.0	2.5	99.8	94.0	3.1	99.8	94.4
13	2 (0.1)	3 (1.4)	60.0	1.3	100	94.0	1.4	100	94.4

*Results estimated from the group used to validate the scoring system (secondary series).

**Figure 3:**
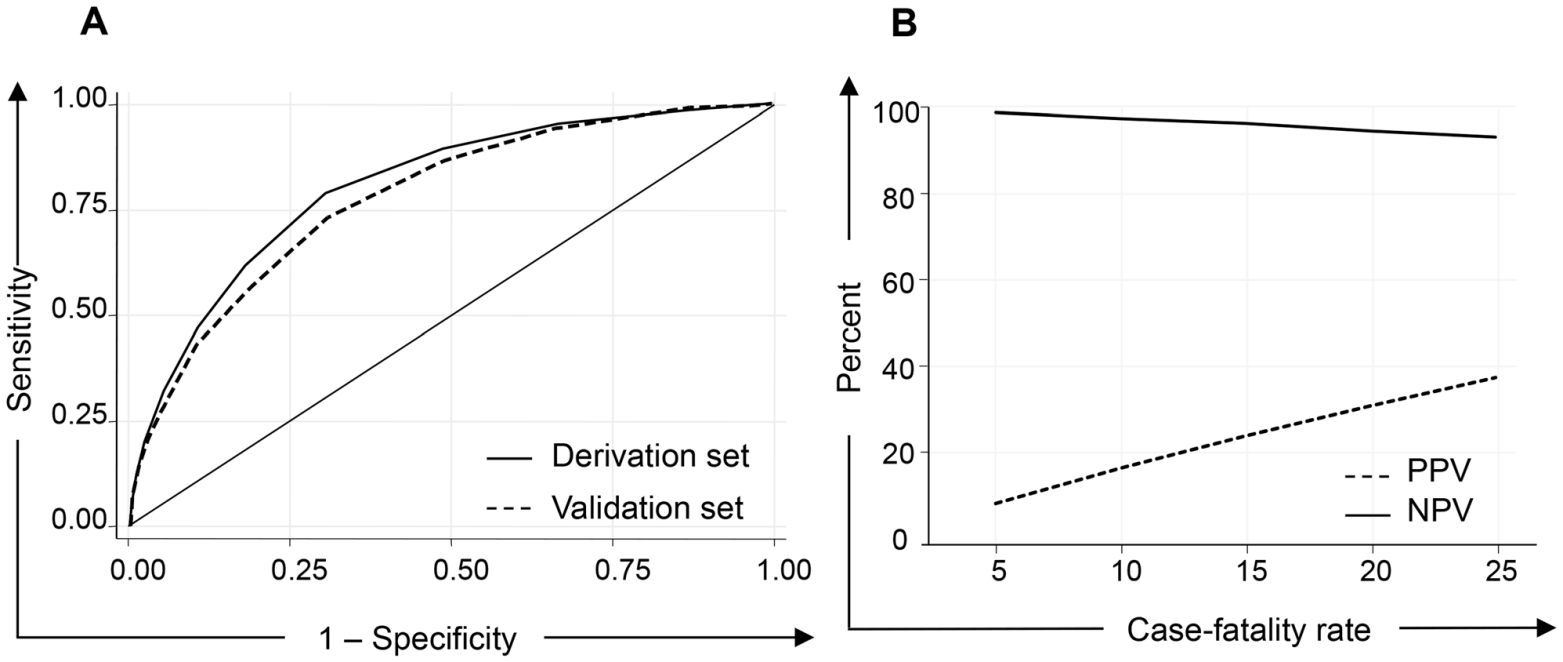
Receiver operating curves (derivation and validation sets) and predictive values of prognostic scoring system. PPV, positive predictive value; NPV, negative predictive value.

The areas under the ROC curves were 0.80 (95% CI 0.78–0.82) for the derivation set and 0.78 (95% CI 0.75–0.81) for the validation set (Fig. 3A). No statistically significant differences were found when comparing derivation and validation curves (p>0.05).

Hence, the prognostic score of 4 was chosen to evaluate the positive and negative predictive values versus several values of probability of death. It was observed that the positive predictive values ranged from 8.8% to 37.9% when the case-fatality rate ranged from 5% to 25%. Negative predictive values ranged from 98.9% to 93.6% (Fig. 3B).

## Discussion

This is the first nationwide study of prognostic factors of death from VL in Brazil. The findings of this investigation contribute greatly to the identification and management of severe VL cases, because a prognostic scoring system for death from VL has been developed and validated. At the time of clinical suspicion of VL, death was reliably predicted by the presence of splenomegaly, edema, weakness, bleeding, jaundice, age (1 year or younger and older than 19 years old), *Leishmania*–HIV co-infection, and presence of bacterial infection. The majority of these risk factors for death can be easily observed by a physician or another health professional in basic health units, allowing early detection of VL cases potentially severe enough for redirection to specialized health services. It is known that correct management of cases by local health services plays an important role in preventing death caused by this disease. According to the clinical point of view and the predictive scoring system proposed here, a patient with a score of 4 or more should have maximum surveillance and attention, because this score presents a risk of death of approximately 4.5% or more. Avoidance of death should be the aim of health care. Hospitalization should be required for all these groups because specific treatment and measures such as hydration, antipyretics, antibiotics, blood therapy, and nutritional support, should be administered and testing to monitor treatment should be performed [12]. According to the clinical point of view and the predictive scoring system proposed here, a patient with a score of 0 or 1 has a low risk of death (<1.1%), indicating that outpatient treatment is potentially safe. Patients with a score of 2 or 3 have a risk of less than 3.2%, which may indicate treatment during a short stay in the hospital or very close follow-up on an outpatient basis. A score of 4 showed high negative predictive value, confirming that patients with a score less than 4 have little chance of death from VL. The positive predictive value was low because of the low cutoff score.

Guidelines indicating clinical recommendations for lethality reduction of VL, including a scoring system used by the leishmaniasis national control program, was published recently. This scoring system was developed using patients from northeast Brazil and clinical and laboratory data; bleeding, edema, jaundice, bacterial infection, and AIDS were also observed to be risk factors associated with death [12]. The two scoring systems differ mainly regarding laboratory data, which are not available in SINAN. Although most predictors identified in this study have been previously recognized as factors of prognostic importance in VL [6], [14], [15], [21], [22], herein we show the magnitude and influence of these factors on prognosis of the death. It is important to emphasize that the proposed score was validated with a different dataset than the one used to estimate the model. Recently, a meta-analysis regarding risk factors for adverse prognosis and death from VL observed that the main limitation of the studies was the absence of validation procedures for the few prognostic models developed so far [22].

Among the factors identified, two (age and bleeding) were the major predictors of death from VL. Patients up to 6 months old and those 65 years or older have a worse prognosis (score 5), followed by those aged older than 50 years to younger than 65 years (score 3). Patients older than 6 months and up to 1 year and those older than 19 years to age 50 years presented lower scores (score 2). According to Brazilian Guidelines for Managing Severe Disease, every VL patient younger than age 6 months or older than 65 years should be considered as having a severe case. Children between the ages of 6 months and 1 year and adults between the ages of 50 years and 65 years should also receive greater attention, because they are more likely to experience progression to severe disease [23]. However, the prospective study by Caldas et al. [24] did not identify differences in clinical and laboratory parameters between children and adults that would indicate different clinical management. Children are generally more susceptible to infectious diseases with relatively higher frequencies of neutropenia and lymphocytosis, because neutrophils are the first-line of defense against bacterial infections [24], [25]. A case-control study conducted in Teresina (northeastern Brazil) revealed that very young children were at higher risk for bacterial infections, diarrhea, and severe anemia, thus contributing to their worse prognosis [6]. Additionally, other authors also observed a poor prognosis in younger children and in the elderly [14], [21], [26], [27].

Some studies show that the presence of bleeding and bacterial infections account for the most fatal complications [6], [14], [16], [21], [28], [29]. Bleeding is part of the coagulopathy associated with the systemic inflammatory response; in VL, thrombocytopenia and hepatic dysfunction contribute to hemorrhagic complications [6]. In severe cases, hemorrhagic phenomena are probably associated with disseminated intravascular coagulation [5], such as the activation of coagulation and fibrinolysis initiated as part of the inflammatory response by a mechanism similar to that established for sepsis [30]. In addition, patients with VL are characteristically neutropenic, and this immunosuppression increases the risk for secondary bacterial infections and other concealed infections, contributing to a higher risk of death [29].

Our data showed that *Leishmania*–HIV co-infection is a factor that worsens the prognosis of VL. In fact, the clinical course and prognosis for these individuals differ from those of non-HIV-infected patients [31]. *Leishmania*–HIV co-infection is characterized by significantly higher drug toxicity, relapse rates, and mortality rates and lower cure rates than for non-HIV-infected individuals with VL [6], [31], [32]. Co-infection exerts a synergistic detrimental effect on the cellular immune response because it targets similar immune cells [33]. The Brazilian Ministry of Health recommends HIV testing for all VL patients and treatment with liposomal amphotericin B for all those who are co-infected [12]. This assures major reliability of this variable in the prognostic scoring system for death.

Several studies have reported that splenomegaly, edema, weakness, and jaundice are associated with worse prognosis [23], [34]. Our score system suggests that these clinical manifestations could predict VL mortality and contribute to better clinical management.

Although VL is a reportable disease in Brazil, the underreporting of cases and deaths with a non-specific clinical picture of disease cannot be ruled out. However, SINAN covers all public and private health care systems and their various levels of complexity. Furthermore, it is noteworthy that the medication used for treatment is solely dispensed by the government, which has minimized underreporting. Despite the improvement in the Brazilian notification systems in the past two decades, several data were missing regarding the final classification of cases (confirmed or discarded) and outcome (cure, death from VL, death by other causes, abandonment of treatment, or transference). These missing data represent a limitation in this study. Although some variables have shown significant differences between the individuals included and excluded from the study, these differences between each category were minimal (up to 3%) and possibly did not compromise our results. These differences occurred because the number of individuals studied was much larger (approximately seven-fold) than the number excluded. However, the need to exclude the patients from the study because of lack of proper registration in SINAN points to the need to improve this information system and the quality of surveillance of VL in Brazil. The variable “time to clinical diagnosis”, defined as the time between the dates of onset of symptoms and notification, was significant only in univariate analysis, in spite of its importance for prognosis of VL patients. The lack of association is likely attributable to recall bias and the difficulty in accurately defining the onset of symptoms. Another possible limitation is that our analyses did not take into account all possible factors that could contribute to unfavorable evolution of VL, such as nutritional status and the presence of other comorbidities (autoimmune diseases, kidney failure, liver or heart diseases, alcoholism, and other drug abuse) and interventions (treatment and clinical management) because these are not collected in the SINAN information system. The miscalibrations found in the scoring system for patients at lowest risk or highest risk (that is, scores 0–1 or 11–13) are a potential limitation; however, they are unlikely to change clinical decision-making.

Despite these limitations, we believe that our prognostic scoring system has good performance and provides relevant information, such as the probability of death according to the signs and symptoms present at the moment of clinical suspicion, thus helping to improve the clinical management of patients with VL. It was developed using all cases recorded throughout the Brazilian territory over the course of 5 years (large sample size), which ensures excellent validity (internal) of the study. Furthermore, by not relying on laboratory results, this scoring system can be used in any health facility, even in the most basic facilities and in those located in more remote areas of the large Brazilian territory. This also allows the possibility of using (and evaluating) this prognostic system in less developed countries with occurrences of VL. Although this study was based on VL cases in Brazil, where *L. infantum* is a common agent of disease, we believe that our scoring system may be useful also in the Old World, where VL is caused by *L. donovani*. Studies conducted in endemic areas in the Old World and in the New World have showed some similar risk factors for death among these species of *Leishmania* [14]–[16], [26]–[28]. However, the scoring system can have poor performance in other populations because of the differences between the characteristics of patients, health care systems and diagnostic methods [18], and species of etiological agents. Therefore, it is necessary to perform external validation of the scoring system in such areas to determinate its predictive performance.

This scoring system should be used in clinical practice after an external validation method. Thus, it is essential to quantify its predictive performance with a new series of patients, ideally in a different location [35], [36]. The present scoring system constitutes the first step in the formulation of a consistent prognostic model that can be improved with the inclusion of other data (e.g., of other comorbidities). As with any predictive score, it should not be used in a definitive manner; clinical decisions should remain dependent on clinical judgment.

### Conclusion

At the time of clinical suspicion of VL, the ability to predict death without the need for laboratory results makes our scoring system simple and useful in health facilities of any degree of complexity. The classification of patients according to their prognosis for death can assist decision-making regarding transference to hospital for care and admission to the intensive care unit, and can maximize surveillance, early detection, and treatment of complications. Therefore, our prognostic scoring system for VL may suggest changes in surveillance strategies to improve the clinical management of severe cases and may contribute to reduction of the case-fatality rate.

## Supporting Information

S1 Supporting Information **STROBE checklist.**
(DOCX)
